# Meditation effects within the hippocampal complex revealed by voxel-based morphometry and cytoarchitectonic probabilistic mapping

**DOI:** 10.3389/fpsyg.2013.00398

**Published:** 2013-07-09

**Authors:** Eileen Luders, Florian Kurth, Arthur W. Toga, Katherine L. Narr, Christian Gaser

**Affiliations:** ^1^Laboratory of Neuro Imaging, Department of Neurology, UCLA School of MedicineLos Angeles, CA, USA; ^2^Department of Neurology, UCLA School of MedicineLos Angeles, CA, USA; ^3^Department of Psychiatry, Jena University HospitalJena, Germany; ^4^Department of Neurology, Jena University HospitalJena, Germany

**Keywords:** cytoarchitectonics, hippocampus, mapping, meditation, mindfulness, MRI, subiculum, VBM

## Abstract

Scientific studies addressing anatomical variations in meditators' brains have emerged rapidly over the last few years, where significant links are most frequently reported with respect to gray matter (GM). To advance prior work, this study examined GM characteristics in a large sample of 100 subjects (50 meditators, 50 controls), where meditators have been practicing close to 20 years, on average. A standard, whole-brain voxel-based morphometry approach was applied and revealed significant meditation effects in the vicinity of the hippocampus, showing more GM in meditators than in controls as well as positive correlations with the number of years practiced. However, the hippocampal complex is regionally segregated by architecture, connectivity, and functional relevance. Thus, to establish differential effects within the hippocampal formation (cornu ammonis, fascia dentata, entorhinal cortex, subiculum) as well as the hippocampal-amygdaloid transition area, we utilized refined cytoarchitectonic probabilistic maps of (peri-) hippocampal subsections. Significant meditation effects were observed within the subiculum specifically. Since the subiculum is known to play a key role in stress regulation and meditation is an established form of stress reduction, these GM findings may reflect neuronal preservation in long-term meditators—perhaps due to an attenuated release of stress hormones and decreased neurotoxicity.

## Introduction

The scientific literature recording anatomical variations in meditators' brains has grown rapidly in recent years (Lazar et al., 2005; Pagnoni and Cekic, 2007; Holzel et al., 2008, 2010; Luders et al., 2009b, 2011, 2012a,b,c; Vestergaard-Poulsen et al., 2009; Grant et al., 2010; Tang et al., 2010, 2012; Murakami et al., 2012; Kang et al., 2013; Leung et al., 2013). Meditation effects, either established as differences between mindfulness practitioners and controls (using cross-sectional designs), as correlates between anatomical measures and the amount of practice, or as actual brain changes due to mindfulness practices (using longitudinal designs), have been observed for numerous cerebral measures. Findings with respect to the attributes of gray matter (GM), however, are amongst the most widely reproduced (Lazar et al., 2005; Pagnoni and Cekic, 2007; Holzel et al., 2008, 2010; Luders et al., 2009b; Vestergaard-Poulsen et al., 2009; Grant et al., 2010; Murakami et al., 2012; Kang et al., 2013; Leung et al., 2013). Moreover, numerous studies have revealed meditation effects in the vicinity of the hippocampus, such as more hippocampal and parahippocampal GM, larger hippocampal dimensions—both globally (total hippocampal volume) and locally (radial hippocampal distances)—as well as enhanced fiber integrity in white matter pathways connecting with the hippocampus (Holzel et al., 2008; Luders et al., 2009b, 2011, 2012c; Murakami et al., 2012; Leung et al., 2013). Altogether, this suggests GM to be a sensitive anatomical marker for determining links between mindfulness practices and brain anatomy, with the hippocampal complex implicated as a structure of particular interest.

To further explore GM characteristics in the framework of meditation, we analyzed a large sample of 100 subjects (i.e., 50 long-term meditation practitioners and 50 control subjects, closely matched for sex, age and handedness). We first applied a standard, whole-brain voxel-based morphometry (VBM) approach and, in accordance with prior studies, revealed significant meditation effects in the vicinity of the hippocampus. According to the matched filter theorem, VBM is most sensitive to effects in the size of the selected smoothing kernel. However, additional effects, ranging above and below that particular spatial scale, may be missed. Similarly, effects that largely deviate from the shape of the applied filter (e.g., occurring in non-spherical structures) may not be captured. Thus, since the hippocampal formation represents a complex of anatomic divisions varying in size and shape, we utilized a second volumetric approach refined by cytoarchitectonic probabilistic mapping to establish differential effects within (peri-) hippocampal subregions.

## Materials and methods

### Subjects

The study included 50 meditation practitioners (28 men, 22 women) and 50 control subjects (28 men, 22 women). Their ages ranged from 24 to 77 years, where groups were closely matched for age [mean ± *SD*: 51.4 ± 12.8 years (meditators) vs. 50.4 ± 11.8 years (controls)]. While scans for the controls were obtained from the International Consortium for Brain Mapping (ICBM) database of normal adults (http://www.loni.ucla.edu/ICBM/Databases/), meditators were newly recruited from various meditation venues in the greater Los Angeles area. Years of meditation experience ranged between 4 and 46 years (mean ± *SD*: 19.8 ± 11.4 years). A detailed overview with respect to each subject's individual practice has been provided elsewhere (Luders et al., 2012a). The majority of subjects (89%) indicated that they were right-handed; six meditation practitioners and five control subjects were left-handed. All subjects gave their informed consent in accordance with the policies and procedures of UCLA's Institutional Review Board.

### Image acquisition and preprocessing

All subjects (i.e., meditators and controls) were scanned at the same site, using the same scanner and image acquisition protocol. Specifically, magnetic resonance images were acquired on a 1.5 T Siemens Sonata scanner (Erlangen, Germany) using an 8-channel head coil and a T1-weighted MPRAGE sequence (1900 ms TR, 4.38 ms TE, 15° flip angle, 160 contiguous sagittal slices, 256 × 256 mm FOV, 1 × 1 × 1 mm voxel). Data was analyzed using SPM8 software (http://www.fil.ion.ucl.ac.uk/spm) and the VBM8 toolbox (http://dbm.neuro.uni-jena.de/vbm.html). Using the same generative model, images were corrected for magnetic field inhomogeneities and tissue-classified into GM, white matter and cerebrospinal fluid. The tissue segmentation procedure was further refined by accounting for partial volume effects (Tohka et al., 2004) and by applying adaptive maximum *a posteriori* estimations (Rajapakse et al., 1997) and non-local means denoising (Manjon et al., 2010). The resulting GM partition was then spatially normalized to the DARTEL template (provided by the VBM8 toolbox) using linear (12-parameter affine) transformation and high-dimensional warping (Ashburner, 2007). This set of warped GM segments in scaled space provided the basis for the standard VBM approach. In addition, we generated GM segments in native space constituting the input for the probabilistic approach.

### Voxel-wise GM (VBM approach): whole brain

As described previously (Luders et al., 2009a), the warped GM segments in scaled space were divided by the non-linear components (but not the linear components) derived from the normalization matrix. This modulation step serves to preserve actual GM values locally, while still accounting for the individual differences in brain size (via proportional scaling). The modulated GM volumes in scaled space were smoothed with a Gaussian kernel of 6 mm full-width-at-half-maximum (FWHM). Using these smoothed scaled GM segments, statistical analyses (described below) were conducted at each voxel across the entire brain.

### Volumetric GM (probabilistic approach): hippocampal complex

This refined approach utilized the three-dimensional (3D) probabilistic labels of the following (peri-) hippocampal subsections: (I) cornu ammonis (CA), (II) fascia dentata (FD), (III) entorhinal cortex (EC), (IV) subiculum (SUB), and (V) hippocampal-amygdaloid transition area (HATA). These 3D labels constitute cytoarchitectonic probabilistic maps, available as part of the Anatomy Toolbox (Eickhoff et al., 2005), that were originally created using cell-body stained histological sections of 10 *post mortem* brains, as detailed elsewhere (Amunts et al., 2005). Briefly, the cytoarchitectonically-defined structures were digitized, warped into MNI single-subject space and converted into 3D probability maps. Thus, each voxel within a 3D probability map contains a count of how many brains (out of ten) had that voxel labeled as the respective hippocampal subregion, therefore coding inter-individual cytoarchitectonic variability in MNI space.

In the current study, these 3D cytoarchitectonic probabilistic maps were first converted from MNI space into each subject's native space. Then, the individual GM segments in native space were multiplied voxel-wise with the 3D probabilistic labels of the five hippocampal substructures, bilaterally. Since the labels encode voxel-wise probabilities ranging between 0 and 100% (rather than a binary value indicating if a voxel is part of the label or not), multiplying them with the GM segments generates probability-weighted voxel-wise GM volumes within each (peri-) hippocampal subregion. The voxel-wise values were added yielding one single GM value per subregion per subject. Using the region-specific probability-weighted volumetric GM (in mm^3^), statistical analyses were conducted as detailed below.

### Statistical analyses

#### VBM approach

Voxel-wise GM differences between meditators and controls were examined, while co-varying for gender and age, via the general linear model in SPM8. In order to avoid possible edge effects, all GM voxels with values of less than 0.1 were excluded (absolute threshold masking). Threshold-free cluster enhancement (Smith and Nichols, 2009) was used to detect significant clusters at *p* ≤ 0.05, corrected for the entire search volume by controlling the False Discovery Rate (Benjamini and Hochberg, 1995). In addition, within the sample of meditators, partial correlation analyses were performed to explore associations between voxel-wise GM and the number of meditation years, while removing the effects of gender and age.

#### Probabilistic approach

Volumetric GM differences between meditators and controls were examined using the general linear model in SPSS20 in a multivariate analysis of co-variance (MANCOVA), with the subregions as dependent measures and gender, age and brain size (approximated by the linear scaling factor derived from the normalization matrix)^1^ as covariates. The significant main effect was followed by *post hoc* comparisons to examine group differences for each subregion separately, where *p* ≤ 0.05 was determined as the threshold for statistical significance. In addition, within the sample of meditators, partial correlation analyses were performed to explore associations between region-specific volumetric GM and the number of meditation years, while removing the effects of gender, age and brain size.

## Results

### VBM analysis

**Figure 1:**
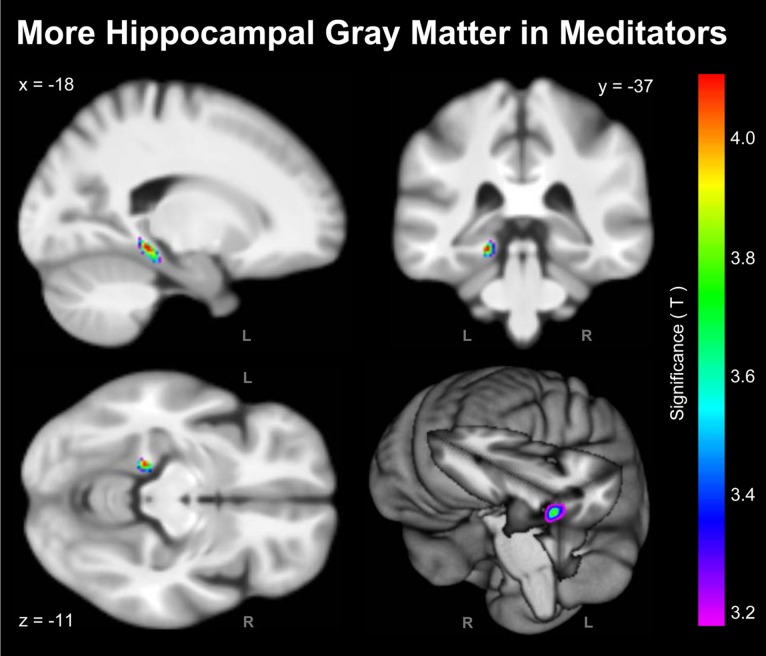
**Significant Group Differences**. Shown are the outcomes of the voxel-wise analysis (VBM approach) indicating more hippocampal GM in meditators compared to controls (L, left hemisphere; R, right hemisphere). For the purpose of illustrating the significance gradient, the color bar encodes the *T*-value at *p* ≤ 0.001, uncorrected. The difference cluster was confirmed when applying corrections for multiple comparisons at *q* = 0.05. Displayed are section views (sagittal, coronal, axial) and the rendered view of the mean brain created from the whole study population (*n* = 100). The *x*-, *y*-, *z*-coordinates in MNI space indicate the significance maximum.

As shown in Figure 1, the voxel-wise analysis revealed one cluster situated in the vicinity of the left hippocampus with significantly more GM in meditators than in controls. The significance maximum was located at *x*; *y*; *z* = −18; −37; −11. When extracting the GM values at the aforementioned peak voxel for each meditator and relating them to the individual number of meditation years, there was a significant positive partial correlation (*r* = 0.34; *p* = 0.015) suggesting that there is an increase of GM with increasing meditation experience.

### Probabilistic analysis

Descriptively, volumetric GM was larger on average in meditators than in controls (Table 1). The multivariate model, including all 10 subregions, yielded a significant main effect of group (*F* = 2.067; *p* = 0.036). Subsequent *post hoc* comparisons of each subregion revealed significantly higher values in meditators within the left subiculum (*F* = 4.126; *p* = 0.045) and right subiculum (*F* = 4.786; *p* = 0.031). In addition, there was a trend for significance for the left hippocampal-amygdaloid transition area (*F* = 3.333; *p* = 0.071). Descriptively, for all subregions, partial correlations between the number of meditation years and volumetric GM were positive. However, effects were relatively small and only the left subiculum reached statistical significance (*r* = 0.327; *p* = 0.025). In addition, there was a trend for significance for the left entorhinal cortex (*r* = 0.271; *p* = 0.066)^2^.

**Table 1:** **Volumetric GM estimates (in mm^3^)**.

**Subregion**	**Group**	**Mean**	**Standard deviation**	**Significance (*p*)**
**LEFT HEMISPHERE**				
CA	MED	4494.95	281.06	0.798
	CTL	4487.48	304.45	
EC	MED	3913.80	324.90	0.817
	CTL	3898.00	352.78	
FD	MED	2320.16	146.09	0.610
	CTL	2309.36	161.30	
HATA	MED	272.38	24.70	0.071
	CTL	264.53	25.02	
SUB	MED	3080.62	179.04	0.045^*^
	CTL	3009.22	218.55	
**RIGHT HEMISPHERE**				
CA	MED	4633.59	268.21	0.281
	CTL	4576.92	370.70	
EC	MED	4272.15	307.80	0.211
	CTL	4193.26	350.94	
FD	MED	2321.77	136.09	0.213
	CTL	2289.41	181.99	
HATA	MED	229.65	19.07	0.287
	CTL	225.98	21.13	
SUB	MED	3279.81	174.46	0.031^*^
	CTL	3199.38	247.18	

*CA, cornu ammonis; EC, entorhinal cortex; FD, fascia dentata; HATA, hippocampal-amygdaloid transition area; SUB, subiculum; MED, meditators; CTL, controls. The asterisks mark the significant group differences (p ≤ 0.05)*.

## Discussion

To our knowledge, this is the first study combining a whole-brain, voxel-based approach with probability-weighted cytoarchitectonic information to assess links between meditation and local GM. With 100 subjects (50 meditators, 50 controls), where meditators have been practicing close to 20 years on average, our sample constitutes one of the largest, more experienced meditation cohorts studied to date. Our study revealed robust meditation effects, with more GM in meditators than in controls within the hippocampal complex as well as positive associations between (peri-) hippocampal GM and number of practice years.

Several studies have demonstrated the hippocampus to be anatomically altered in meditation practitioners, with more hippocampal or parahippocampal GM (Holzel et al., 2008; Leung et al., 2013), larger hippocampal volumes globally (Luders et al., 2009b, 2012c), larger hippocampal radial distances locally (Luders et al., 2012c), as well as enhanced fiber integrity in white matter pathways connecting with the hippocampus (Luders et al., 2011). Moreover, positive correlations have been reported between parahippocampal GM and a specific facet of mindfulness (Murakami et al., 2012). In addition, functional experiments, either using positron emission tomography (PET) or functional magnetic resonance imaging (fMRI), seem to implicate the hippocampus and/or the parahippocampal gyrus as significantly involved in meditation processes (Lou et al., 1999, 2005; Lazar et al., 2000; Holzel et al., 2007; Engstrom et al., 2010; Kalyani et al., 2011). The observed voxel-wise findings in the vicinity of the hippocampus are in close agreement with these previously reported findings. The hippocampal complex, however, is not a single, homogeneous structure but comprises of cytoarchitecturally distinct subdivisions. Thus, in contrast to previous analyses where the hippocampus was mostly considered as a single unit (either already defined *a priori* or detected as different *a posteriori*), we set out to discriminate between the different subregions of the hippocampal complex. While the borders of (peri-) hippocampal subsections rarely match macro-anatomical landmarks, microscopic labeling enables a more precise localization of these boundaries (Amunts et al., 2005). We therefore analyzed local GM within 3D (peri-) hippocampal labels that were carefully determined using microscopic information obtained via silver staining for cell bodies (Amunts et al., 2005). This unique approach revealed significant meditation effects within the left and right hippocampal subiculum.

The hippocampus is a key structure for memory processes, both in terms of memory encoding and retrieval, and the subiculum seems to occupy a central position within this memory system (Naber et al., 2000). Our current findings might thus account for positive effects of meditation on memory performance, such as an enhanced capacity of working memory as well as increased specificity of autobiographical memory (Williams et al., 2000; Heeren et al., 2009; Kozhevnikov et al., 2009; Chiesa et al., 2011). With particular respect to the subiculum, a specific association with episodic recollection has been reported (Viskontas et al., 2009), suggesting that the subiculum plays a significant role for “re-experiencing an event as part of one's personal past.” Regular meditating, especially meditation over many years, involves engaging in an established routine. It not only leads to a variety of mind-altering / new states, but it also involves re-experiencing states, such as the shift that occurs from normal consciousness to meditation (Travis and Wallace, 1999). Our current findings of GM alterations within the subiculum might thus be associated with this kind of recollective experience during meditation. Alternatively, or as a complementary association, more (peri-) hippocampal GM might also account for meditators' abilities to habitually engage in mindful behavior by regulating their responses in a context-sensitive fashion. The key role of the subiculum within the behavioral inhibition system has been extensively reviewed (McNaughton, 2006). One of its functions is to compare and integrate incoming goal-related information and to produce output when conflict between incompatible goals is detected. Thus, more GM within the subiculum might be the neuronal foundation that allows meditators to mindfully choose from an array of behavioral options leading to well-adjusted responses not only to everyday occurrences, but also major life events. This ability is often referred to as non-reactivity.

Due to the cross-sectional design of our study, it is impossible to determine if the observed GM alterations are a prerequisite for meditation practices or an actual consequence of meditating. More specifically, it is possible that more GM in the hippocampal subiculum constitutes an innate brain characteristic that attracts an individual toward meditation. Likewise, a specific neuronal build-up may facilitate reaching desired states during meditation and/or experiencing positive effects following meditation, and may thus help maintaining a regular and long-term practice via intrinsic positive reinforcement. On the other hand, engaging in meditation is an active mental process that, depending on the technique, incorporates efforts to exercise awareness, attention, concentration, focus, etc. Thus, if occurring regularly, such intense mental processes may directly affect local GM volume by initiating microscopic events, such as dendritic branching, synaptogenesis or even adult neurogenesis as occurs in the dentate (Eriksson et al., 1998; Gage, 2002; Elder et al., 2006). Although the precise underlying mechanisms for GM changes in healthy volunteers are still poorly understood, similar training-induced increases of hippocampal GM, albeit unrelated to meditation, have been reported during periods of extensive learning and exercise (Draganski et al., 2006; Pereira et al., 2007; Boyke et al., 2008). Alternatively, or as a complementary mechanism to the aforementioned direct training effects, meditation might also have indirect preservative and/or restorative effects by positively affecting stress regulation. It is well-known, for example, that mindfulness-based techniques are highly effective in stress reduction, both in terms of subjectively perceived stress as well as objectively measured biomarkers of stress (Holzel et al., 2010; Jung et al., 2010, 2012; Mohan et al., 2011; Baer, 2003; Ciesla et al., 2012; Jensen et al., 2012; Marchand, 2012; Fan et al., 2013). The hippocampus and especially the subiculum play a major role in stress regulation by inhibiting the hypothalamo-pituitary-adrenocortical (HPA) axis. Specifically, the output neurons of the hippocampal subiculum (i.e., where main effects occurred in the present study) are heavily involved in attenuating the release of stress-induced glucocorticoids (Herman and Mueller, 2006). Prolonged glucocorticoid elevations, in turn, have been demonstrated to increase hippocampal vulnerability to neurotoxicity (Conrad et al., 2007), which may manifest as loss of (peri-) hippocampal neurons or disturbances in hippocampal neurogenesis. Consequently, if actively meditating is accompanied by stress reduction (and thus by an attenuated release of stress hormones and decreased neurotoxicity), meditation practitioners might have more GM in the vicinity of the hippocampus due to neuron preservation and/or neurogenesis. In strong support of this theory, a recent longitudinal VBM study, designed to identify brain regions that changed in association with participation in an 8-week mindfulness-based stress reduction (MBSR) intervention, revealed significant GM increases within the left hippocampus (Holzel et al., 2011).

## Future research

The current findings may suggest that actively meditating induces changes of (peri-) hippocampal GM (either directly or indirectly). However, as our study is cross-sectional in nature, any conclusions with respect to a causal role of meditation remain speculative. Further research incorporating longitudinal designs is clearly needed. Moreover, future studies may also obtain measures of cortisol (a biological marker for stress). This will serve to elucidate the underlying mechanisms for the observed effects by comparing cortisol levels between expert meditators, novice meditators, and non-meditators, by examining possible changes in cortisol levels due to intense periods of meditation, and by relating cortisol levels to structural brain imaging measures. Last but note least, future studies collecting subject-specific information with respect certain lifestyle choices—including diet, recreational drugs, and physical exercise—may further advance this field of research by taking into account additional features (i.e., aside from the actual practice of meditation) discriminating between meditators and non-meditators.

### Conflict of interest statement

The authors declare that the research was conducted in the absence of any commercial or financial relationships that could be construed as a potential conflict of interest.

## Footnotes

^1^While total intracranial volume (TIV) might be a more commonly used measure, co-varying for the linear scaling factor makes the outcomes resulting from the two approaches more comparable. Nevertheless, we also repeated the volumetric GM analysis co-varying for TIV (rather than for the linear scaling factor). However, since both values are highly correlated (*r* = 0.91, *p* ≤ 0.001), the outcomes are very similar (see Results).

^2^When co-varying for TIV (rather than for the linear scaling factor), the main effect was significant with *F* = 2.013 (*p* = 0.041). The *post hoc* comparisons revealed *F* = 4.010 (*p* = 0.048) for the left subiculum, *F* = 4.804 (*p* = 0.031) for the right subiculum, and *F* = 3.468 (*p* = 0.066) for the left hippocampal-amygdaloid transition area. The partial correlations were *r* = 0.314 (*p* = 0.032) for the left subiculum and *r* = 0.257 (*p* = 0.082) for the left entorhinal cortex.
